# Cryptogenic stroke as a working diagnosis: the need for an early and comprehensive diagnostic work-up

**DOI:** 10.1186/s12883-023-03206-6

**Published:** 2023-04-14

**Authors:** Maurizio Acampa, Pietro Enea Lazzerini, Simona Lattanzi, Marta Rubiera

**Affiliations:** 1 Stroke Unit, Department of Emergency-Urgency and Transplants, Azienda Ospedaliera Universitaria Senese, “Santa Maria alle Scotte” General-Hospital, Siena, Italy; 2 Department of Medical Sciences, Surgery and Neurosciences, University of Siena, Siena, Italy; 3 Department of Experimental and Clinical Medicine, Neurological Clinic, Marche Polytechnic University, Ancona, Italy; 4 Stroke Unit, Department of Neurology, Hospital Vall d’Hebron, Barcelona, Spain; 5 U.O.C. Stroke Unit, Policlinico ‘S. Maria alle Scotte’, viale Bracci, n.1, Siena, 53100 Italy

**Keywords:** Atrial cardiopathy, Cryptogenic stroke, Nonstenosing atherosclerosis, Noncardioembolic stroke, Atrial cardiopathy, Implantable cardiac monitor, Atrial fibrillation

## Abstract

In the Nordic Atrial Fibrillation and Stroke (NOR-FIB) study, the causes of ischemic stroke were identified in 43% of cryptogenic stroke patients monitored with implantable cardiac monitor (ICM), but one-third of these patients had non-cardioembolic causes. These results suggest the need for an early and comprehensive diagnostic work-up before inserting an ICM.

The recent European Stroke Organisation (ESO) guidelines [1] recommend early and prolonged electrocardiogram (ECG) monitoring with an implantable cardiac monitor (ICM) following an ischemic stroke or transient ischaemic attack (TIA) of undetermined origin to identify subclinical atrial fibrillation (AF). Detecting subclinical AF in cryptogenic stroke (CS) can be particularly useful, as it results in an increased rate of anticoagulation initiated and may reduce the risk of stroke recurrence [2]. The Nordic Atrial Fibrillation and Stroke (NOR-FIB) study by Ratajczak-Tretel et al., recently aimed to detect and quantify underlying AF in patients with CS or TIA using ICM, to optimise secondary prevention, and to test the feasibility of ICM usage for stroke physicians. The results of this study confirmed the relevance of prolonged cardiac monitoring in CS, showing paroxysmal AF in 28.6% of patients (detected early in 86.5% of patients after ICM insertion) with anticoagulants usage in 97.3% of AF patients at 12-month follow-up [3].

In *BMC Neurology*, the same authors provide new results from their prospective, multicentre, international, observational, real-life study [4]. They show that the causes of ischemic stroke were identified in 43% of CS patients monitored with ICM, but, surprisingly, one-third of these patients had non-cardioembolic causes, including large-artery atherosclerosis, small vessel disease and hypercoagulable states.

These data raise relevant issues surrounding the need for an early and comprehensive diagnostic work-up to avoid unnecessary and costly monitoring with ICM that in specific cases could also be of uncertain clinical relevance.

In recent years, previous studies [5, 6] showed the higher prevalence of non-stenotic but high-risk atherosclerotic plaques ipsilateral to acute brain infarction compared to the contralateral, infarct-free side among patients with CS, suggesting the need to reclassify the etiology of up to 15% of strokes [6]. Specifically, in CS, new advancements in vessel wall magnetic resonance imaging (MRI) could provide more details about atherosclerotic plaques beyond the degree of luminal stenosis. Allowing careful characterization of plaque components and identifying potential imaging markers of vulnerable plaques, such as intraplaque haemorrhage, lipid rich necrotic core, and thin or ruptured fibrous caps, would enable identification of culprit plaques [7].

Furthermore, the role of neuroimaging is crucial to exclude small vessel disease before starting cardiac monitoring, and, in specific cases, MRI can be a more reliable diagnostic method than a brain computed tomography (CT) scan [8]. For example, a recent study [9] in patients with stroke attributed to large- or small-vessel disease found that monitoring with an ICM detected significantly more AF events over 12 months. However, it is not clear whether identifying AF in these patients could be considered as a pathogenic, underlying mechanism related to the initial stroke.

The ESO guidelines [1] also highlighted the limited predictive value of some potential blood, echocardiographic, and ECG biomarkers, and suggested avoiding their use for excluding patients from long-term ECG monitoring. Pre-selection of CS patients with the highest probability of AF could improve the efficiency of monitoring in countries where the healthcare system cannot afford ICM for all CS patients.

Some clinical scores (e.g., HAVOC, Brown ESUS-AF, C2HEST) used together with specific ECG and echocardiographic markers (P wave dispersion, PTFV1, P wave axis, atrial size) are suggested to be useful tools and less expensive alternatives to better guide the diagnostic process [10, 11], and can enable identification of the subset of patients at higher risk of AF. In particular, a HAVOC score ≥ 4 points can predict higher yield of AF detection among CS patients [12], while a C2HEST score ≥ 4 can predict incident of AF in poststroke patients [13]. In contrast, the Brown ESUS-AF score is based on both age and left atrial enlargement, and scores ≥ 2 can help identify CS patients with high risk of occult AF [14]. Furthermore, some ECG markers, such as P wave terminal force in V1 > 5000µV·ms [15, 16], P wave dispersion > 40 ms, and an abnormal P wave axis [17, 18], could help to identify CS patients with specific phenotypes associated with atrial cardiopathy. Not only is atrial cardiopathy often the substrate favoring the occurrence of AF, but it is also the possible independent cause of cardioembolism, therefore directly increasing the risk of atrial thrombosis [19]. However, the prognostic utility of these markers is still unclear and should be prospectively assessed with AF detection and recurrent stroke as outcomes [20]. Furthermore, additional work is needed to standardize measurement of ECG markers, confirm their reliability and predictive value, and define the risk-benefit ratio of specific interventions in high-risk individuals [21]. For these reasons, we cannot yet consider these parameters reliable and robust. In addition, further studies should also include the efficacy of a multimodal approach combining clinical factors, electrocardiography, and biological markers to select CS patients for prolonged cardiac rhythm monitoring.

Currently, it could be advisable to perform a complete diagnostic work-up, including more advanced investigations to identify non-cardioembolic causes (e.g., non-stenotic atherosclerosis, small vessel diseases, hypercoagulable states, occult neoplasms) in patients with negative markers of atrial cardiopathy, before initiation of prolonged cardiac monitoring with an ICM.

## Data Availability

Not applicable.

## Abbreviations

ESO: European Stroke Organisation
ICM: implantable cardiac monitor
AF: atrial fibrillation
CS: cryptogenic stroke
